# Laser Capture Microdissection Coupled Capillary Immunoassay to Study the Expression of PCK-2 on Spatially-Resolved Islets of Rat Langerhans

**DOI:** 10.3390/pharmaceutics13060883

**Published:** 2021-06-15

**Authors:** Shashank Pandey, Zdenek Tuma, Tereza Smrhova, Miroslava Cedikova, Tereza Macanova, Magdalena Chottova Dvorakova

**Affiliations:** 1 Department of Pharmacology and Toxicology, Faculty of Medicine in Pilsen, Charles University, Alej Svobody 1655/76, 32300 Pilsen, Czech Republic; 2 Biomedical Center, Faculty of Medicine in Pilsen, Charles University, Alej Svobody 1655/76, 32300 Pilsen, Czech Republic; zdenek.tuma@lfp.cuni.cz (Z.T.); Miroslava.Cedikova@lfp.cuni.cz (M.C.); magdalena.dvorakova@lfp.cuni.cz (M.C.D.); 3 Department of Physiology, Faculty of Medicine in Pilsen, Charles University, Alej Svobody 1655/76, 32300 Pilsen, Czech Republic; terezasmrhova@seznam.cz; 4 Department of Biology, Faculty of Medicine in Pilsen, Charles University, Alej Svobody 1655/76, 32300 Pilsen, Czech Republic; Tereza.Macanova@lfp.cuni.cz

**Keywords:** PCK2, Langerhans islets, laser capture microdissection, capillary Western blotting, multiplexing

## Abstract

The platform for precise proteomic profiling of targeted cell populations from heterogeneous tissue sections is developed. We demonstrate a seamless and systematic integration of LCM with an automated cap-IA for the handling of a very small-sized dissected tissues section from the kidney, liver and pancreatic Langerhans islet of rats. Our analysis reveals that the lowest LCM section area ≥ 0.125 mm^2^ with 10 µm thickness can be optimized for the detection of proteins through LCM-cap-IA integration. We detect signals ranging from a highly-abundant protein, β-actin, to a low-abundance protein, LC-3AB, using 0.125 mm^2^ LCM section from rat kidney, but, so far, a relatively large section is required for good quality of results. This integration is applicable for a highly-sensitive and accurate assessment of microdissected tissue sections to decipher hidden proteomic information of pure targeted cells. To validate this integration, PCK2 protein expression is studied within Langerhans islets of normal and diabetic rats. Our results show significant overexpression of PCK2 in Langerhans islets of rats with long-term diabetes.

## 1. Introduction

The last several years have seen rapid development of technologies and methods that permit a detailed analysis of the genome and proteome. Even experimental approaches that utilize single-cell analysis are an effective means to understand how cell networks work in concert to coordinate a response at the population level. This interdisciplinary frontier of analytical chemistry, biology, and medicine has provided a unique platform to study a certain type of specific cells in the population to identify heterogeneity between cells. Moreover, organs and tissues consist of multiple cell types. Isolation of particular cell types (structures) can provide insight into (patho) physiological processes in these cells. LCM enables careful dissection of specific cells from tissues that have been snap-frozen. However, one of the most important requirements for the use of LCM samples is the possibility of downstream processing for proteomic and genomic analysis. Various molecular biological techniques have been combined with LCM, such as next-generation sequencing [1], or microarray and quantitative RT-PCR [2,3]. These advancements in the genomic revolution have increased the interest of researchers to combine LCM with proteomics to discover new information [1,4,5,6,7,8,9]. The major obstacles in proteomic studies are their variability from gene products and their inability to self-replicate. Using a technique analogous to PCR to induce in vitro amplification of proteins is impossible. Therefore, developing a methodology for processing a low amount of sample has paramount importance in proteomics.

State-of-the-art systems have demonstrated that the methodology could be optimized for measuring hundreds of proteins from single cell-sized samples using mass spectrometry [10]. Hughes et al., 2014, have demonstrated that scWestern analysis could be used for quantification and multiplexes of up to 11 protein targets per single cell with detection thresholds of <30,000 molecules [11]. Zhu et al., 2018, demonstrated that the NanoPOTS platform can minimize sample processing volume up to <200 nL and can enhance the recovery rate [12]. Furthermore, integration of NanoPOTS with ultrasensitive liquid chromatography-MS allows identification of ~1500 to ~3000 proteins from ~10 to ~140 cells, respectively. Budnik et al., 2018 have developed the SCoPE-MS method to quantify more than 1000 proteins in differentiating mouse embryonic stem cells [13].

As mentioned above, many efforts have been made in the last decade to develop an advanced method for protein quantification using a variety of interdisciplinary approaches. However, quantification of proteins in a very small sample remains a challenge. Many factors are involved when selecting the best option for proteomics, and the decision also depends upon individual approaches from institution to institution. A single approach cannot be fitted in every lab. In this study, we demonstrate for the first time, a seamless and systematic integration of LCM with automated cap-IA to study the expression of phosphoenolpyruvate within microdissected sections of Langerhans islets.

The synthesis of phosphoenolpyruvate (PEP) is an absolute requirement for gluconeogenesis from mitochondrial substrates. Two isoforms of enzyme phosphoenolpyruvate carboxykinase (PEPCK or PCK) could be involved in this process; PEPCK-C present in cytosol (also called PCK1) and mitochondrial PEPCK-M, also called PCK2. PCK1 plays a regulatory role in several cataplerotic processes including e.g., gluconeogenesis, and its function has already been very extensively studied [14]. Contrary, information concerning PCK2 is still very limited. Interestingly, pancreatic β-cells do not exhibit the presence of PCK1 but only PCK2 [15]. Therefore, PCK2 is responsible for PEP production in pancreatic β-cells located within Langerhans islets. The fact that PEP is associated with insulin production indicates an association between PCK2 and insulin secretion [15,16].

To determine, whether long-term type 2 diabetes mellitus affects PCK2 within Langerhans islets, the measurement of the proteomic level of PCK2 was done. Since the islets of Langerhans containing β-cells are unevenly distributed in the pancreas, laser capture microdissection (LCM) was used to collect Langerhans islets from pancreatic tissue to obtain samples containing a comparable amount of β-cells. These samples were very small, so, first, a new methodological approach to analyze such a small amount of input material had to be developed. To our knowledge, this is the first report which describes the integration of LCM with cap-IA using a multiplexing approach.

## 2. Material and Methods

### 2.1. Statement on Welfare of Animals

All experiments were approved by the University Committee for Experiments on Laboratory Animals and Ministry of Education, Youth and Sports of the Czech Republic (MSMS-10669/2016-6; 15 March 2016) and were conducted in accordance with the “Guide for the Care and Use of Laboratory Animals” (NIH Publication No. 85-23, revised 1996) as well as the relevant Guidelines of the Czech Ministry of Agriculture for scientific experimentation on animals.

### 2.2. Animal Specification

Adult male Zucker diabetic fatty (ZDF) rats and age-matched lean rats (controls) were used (*n* = 12) with a mean body weight of about 235 ± 3.4 g (mean ± SEM) for controls and 408 ± 12.6 g for ZDF rats. The animals were housed two per cage and had free access to drinking water. They were fed ad libitum with the Purina 5008 diet (Charles River Laboratories International, Inc., Wilmington, MA, USA) from 12 week of age, which induced type 2 diabetes mellitus and decreased production of insulin in ZDF animals but had no such effect on controls.

Rats were sacrificed by decapitation 34 weeks after the initiation of feeding with the Purina 5008 diet. Organs (heart, kidney and pancreas) were rapidly excised; rinsed with ice-cold saline solution; freed of connective tissue and fat and directly frozen in liquid nitrogen (for protein isolation) or embedded in optimum cutting temperature compound (Takara, Mountain View, CA, USA) and frozen in precooled isopentane (for laser capture microdissection). Samples were kept at −80 °C until use.

### 2.3. Preparation of LCM Section

Frozen rat tissues were cut into 10 µm thick sections using a Leica 1900E cryostat (Leica, Bensheim, Germany). Sections were placed onto special slides designed for laser cutting (MMI, Eching, Munich, Germany). Tissues were stained with alum hematoxylin solution for 8 min and washed by water for 8 min. Finally, the slides were dehydrated with 70% ethanol for 40 s and 100% ethanol for 40 s, subsequently air-dried. After drying, the slide was placed on an inverted microscope. This staining was used because it does not interfere with RNA as well as protein analyses [17,18].

In this study, we used an Olympus IX71 (MMI, Glattbrugg, Zurich, Switzerland), an inverted microscope, comprised of a UV laser. A very low laser pulse energy (<1 µJoule was used, which leaves the target material unaffected with no impact on subsequent protein analysis. The optimized the laser setup was: (a) cut velocity or speed: 15 µm/s; (b) laser focus: 54.4%; (c) laser power: 95%. We have used MMI-membrane slides PET- membrane 1.4 µm (MMI, Eching, Munich, Germany). The different types of samples can be placed directly on the membrane, which is then inverted and a standard glass slide placed below as per standard protocols.

Under the supervision of a histologist, Langerhans islets were selected and dissected from the slides. In order to collect predominantly β-cells but not α-cells, we have omitted the cutting of a border area for each Langerhans islet [19]. For each rat, Langerhans islets were pooled until the total section area reached more than 0.8 mm^2^ and subsequently, protein or RNA was isolated. For protein isolation, WES buffer (0.5×) combined with fluorescent master (4:1) were added to maintain a dissected area of Langerhans islets 0.05 mm^2^/µL. It corresponds to the final LCM section area 0.25 mm^2^ in 5 µL of loading sample volume. Samples were loaded onto three separate capillaries. Anti-PCK2 Abs at dilution 1:75 were mixed with anti-β-actin Abs dilution 1:75, and used for the analysis. The assay plate was loaded with biotinylated ladder, samples, primary antibodies, HRP-conjugated secondary antibodies and chemiluminescent substrate in the designated wells.

For RNA isolation, RNeasy Micro Kits (Qiagen, Hilden, Germany) was used according to the manufacturer’s instructions. Obtained RNA was immediately reverse transcribed using Superscript III Reverse Transcriptase (Invitrogen, Waltham, MA, USA) for 50 min at 42 °C. The qPCR analysis was done as described previously [3]. The primers were designed to amplify the sequence corresponding to nucleotides1420–1620 (forward: TGCCCATCGAAGGCATCATT; reverse: ACTTGCCGAAGTTGTAGCCA) of the published rat PCK1 cDNA sequence (Genbank Accession No. NM_198780.3), nucleotides 1421–1586 (forward: AGAAGGTGTTCCAATTGATGCC; reverse: ATCGTGCATAATGGTCTTTCCC) of the published rat PCK2 cDNA sequence (Genbank Accession No. NM_001108377.2), and nucleotides 821–1029 (forward: TTCCTTCCTGGGTATGGAATC; reverse: GTTGGCATAGAGGTCTTTACGG) of the published rat β-actin cDNA sequence (Genbank Accession No. NM_031144).

Real-time PCR was performed in the iCycler (Bio-Rad, Prague, Czech Republic). Final assay volumes were 16 μL and contained 3.83 μL of ultrapure water, 8 μL iQ SYBR Green Supermix (Bio-Rad, Prague, Czech Republic), 4 μL of diluted cDNA, and 0.17 μL of each primer (20 nmol/L). The quantitative PCR reactions were performed as follows: denaturation at 95 °C for 10 min followed by 45 cycles of amplification (95 °C for 20 s, 60 °C for 20 s and 72 °C for 20 s). Each run was completed with a melting curve analysis in order to confirm the specificity of amplification and lack of primer dimers. Each pair of primers yielded a single peak in the melting curve and a single band of the expected size in agarose gel. Standard curves were generated for each pair of primers using three-fold serial dilution of cDNA obtained by reverse transcription of RNA from the kidney, the tissue with high expression of PCK1 as well as PCK2. Blank controls with the omitted template were used. In order to test, whether a sufficient amount of material to proceed evaluation was collected, the expression of β-actin was measured in the samples. Only samples showing the presence of β-actin mRNA were used for PCK1 and PCK2 analyses.

### 2.4. Traditional Western Blotting (tradWB)

Western blotting analysis was performed using the Bio-Rad V3 workflow (Bio-Rad, Hercules, CA, USA). We separated 1 µg of recombinant PCK2 on Criterion TGX Stainfree polyacrylamide gel and transferred it onto a PVDF membrane (Trans-Blot Turbo Midi PVDF Transfer Pack; all Bio-Rad) by semidry transfer (25 V, 2.5 A, 7 min). The membranes were blocked in TBS buffer (0.15 M NaCl, 20 mM TRIS–HCl, pH 7.5) with 0.1 % (*v*/*v*) Tween-20 and 5 % (*w*/*v*) nonfat dry milk and incubated overnight with primary antibody (rabbit polyclonal anti-PCK2 antibody, ab70359, dilution 1:1000; Abcam, Cambridge, UK). PVDF membranes were incubated for 90 min with secondary antibody (goat anti-rabbit IgG-HRP, A16116, dilution 1:4000; Thermo Fisher Scientific, Waltham, MA, USA). Detection was performed with Westernbright ECL chemiluminescent reagent (Advansta, Menlo Park, CA, USA) and images were acquired using a ChemiDoc MP Imager (Bio-Rad, Hercules, CA, USA). Images of gels and membranes were processed with Imagelab 6.0.1 software (Bio-Rad, Bio-Rad, Hercules, CA, USA). The total run time that includes all steps from the gel running to the chemiluminescent signal acquisition was 20.5 h.

### 2.5. LCM Samples for Kidney, Pancreas, and Heart Tissues

Frozen rat tissues were cut into 10 µm thick sections and stained with alum hematoxylin as per the protocol mentioned above in Section 2.3. For every tissue, a homogenous place was selected and four different sections of area 0.25 mm^2^ were cut. Each LCM section was dissolved in 5 µL of prepared WES buffer mix (0.5× WES buffer combined with fluorescent master). Samples were heated at 100 °C for 10 min followed by centrifugation at 1000× *g* for 60 s at room temperature. Sections of similar tissue origin were pooled together before final loading into capillary. We loaded 5 µL of the sample per capillary with three technical replicates.

### 2.6. Preparation of Langerhans Islets

Total, six diabetic rats (*n* = 6) and six control rats (*n* = 6), were used for the study. Frozen rat pancreatic tissues were cut into 14 µm thick tissue sections and stained with alum hematoxylin as per the protocol mentioned above in Section 2.3. 

Under the supervision of a histologist, Langerhans islets were selected and dissected from the slides. We collected approx. 40 to 50 islets for each sample containing a total area of approximately 0.8 mm^2^ of the tissue section. The size of islets varies from 0.01 mm^2^ to 0.25 mm^2^. However, the size of the dissected island greatly depends on what part of the island is in a given section, whether it is peripheral or whether the island is cut through the center. For each rat, Langerhans islets were pooled until the total section area reached more than 0.8 mm^2^. WES buffer (0.5×) combined with fluorescent master were added to maintain the dissected area of Langerhans islets 0.05 mm^2^/µL. It corresponds to the final LCM section area 0.25 mm^2^ in 5 µL of loading sample volume. Samples were loaded onto three separate capillaries. Anti-PCK2 Abs at dilution 1:75 were mixed with anti-β-actin Abs dilution 1:75, and used for the analysis. The assay plate was loaded with biotinylated ladder, samples, primary antibodies, HRP-conjugated secondary antibodies and chemiluminescent substrate in the designated wells.

### 2.7. Data Analysis of PCK-2 in Langerhans Islets

Each sample was analyzed in three technical replicates using Compass for SW, version 4.0.0. Compass for SW is the data analysis application for Simple Western instruments, peak areas of PCK2 peak (at 63 kDa) and ACTB peak (at 48 kDa) were extracted for each technical replicate. The normalized PCK2 signal in each replicate was expressed as a ratio of peak areas PCK2 to ACTB. The average of PCK2/ACTB from three technical replicates was calculated for each animal. PCK2/ACTB values were averaged across the healthy and diabetic groups and the difference between the healthy and diabetic groups was tested using a *t*-test.

### 2.8. Capillary Western Blotting (cap-IA)

WES buffer, fluorescent master mix, separation capillaries, and assay plates were purchased from ProteinSimple (San Jose, CA, USA). The primary antibody for β-actin (bs0061R) was purchased from Bioss (Woburn, MA, USA), and the primary antibody for mitochondrial phosphoenolpyruvate carboxykinase PCK2 (ab70359) was purchased from Abcam (Cambridge, UK) and the primary antibody for LC3AB (4108S) was purchased from Cell signaling technologies, Danvers, MA, USA). A secondary antibody was purchased from ProteinSimple. Data analysis of Wes^®^ was performed using “Compass for SW” Version: 4.0.0 Build ID: 0815 designed by ProteinSimple.

Capillary western analyses were performed using Wes^®^ System (WS-3127, ProteinSimple, San Jose, CA, USA). LCM samples were dissolved in WES Buffer (0.5×) combined with 5× fluorescent master mix. Samples were heated at 100 °C for 10 min followed by centrifugation at 1000× *g* for 60 s at room temperature. The fluorescent master mix contains three fluorescent proteins that act as a “ruler” to normalize the distance for each capillary because the molecular weight ladder is only on the first capillary and each capillary is independent. After this denaturation step, primary antibody dilution of different antibodies were added on the plate such as β-actin (1:25, 1:75, 1:100, 1:150, 1:300) or LC3AB (1:100) or mixed dilution of β-actin (1:75) with PCK2 dilution (1:10, 1:25, 1:75). The assay plate was loaded with 5 µL of biotinylated ladder, 5 µL of samples, 10 µL of primary antibodies, 10 µL of HRP-conjugated secondary antibodies and 15 µL of the chemiluminescent substrate in the designated wells. A biotinylated ladder provided molecular weight standards for each assay. After plate loading, the separation of protein and immune detection steps took place in the fully-automated capillary system with a total run time of ~3 h.

## 3. Results

### 3.1. Integration of cap-IA and LCM to Maintain Linearity, Reproducibility, and Accuracy

For successful analysis, it was necessary to obtain a non-saturated, linear, and reproducible signal. A systematic approach was used to achieve LCM-cap-IA integration. Frozen rat kidney tissue of 10 µm thickness was stained and used for laser cutting. A homogenous part of the tissue was selected and the different sizes of LCM sections were cut (Supplementary Figure S1).

A calibration graph was prepared by using tissue lysate of known concentration and proteins extracted from different LCM sections (0.5 mm^2^, 0.25 mm^2^, 0.125 mm^2^, and 0.06 mm^2^) from rat kidney were extracted and observed on 10% SDS-PAGE (Supplementary Figure S2d). After the running of gel, the image was analyzed on Image Lab™ 6.0.1 Software. The sum of all protein bands density in the lane for each standard was estimated and used for creating a linear curve between tissue lysate concentrations used as a standard versus the density measured (Supplementary Figure S2). The graph was used to calculate the total protein concentration of extracted LCM sections of different areas (Supplementary Table S1).

**Figure 1:**
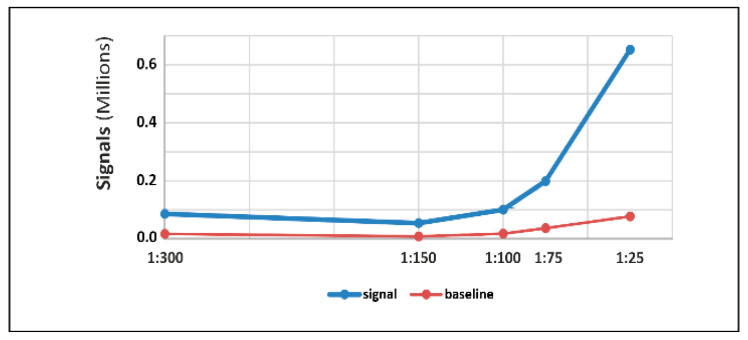
Optimization of anti-β-actin antibodies dilution on capillary western blot: Different dilutions of anti-β-actin (1:25; 1:75; 1:100; 1:150 and 1:300) were used on 0.5 mm^2^ LCM section of rat kidney. Signal and baseline, the area under the curve was analyzed to select the best dilution of anti-β-actin antibodies. Dilution 1:75 was selected and used for further experiments.

**Figure 2:**
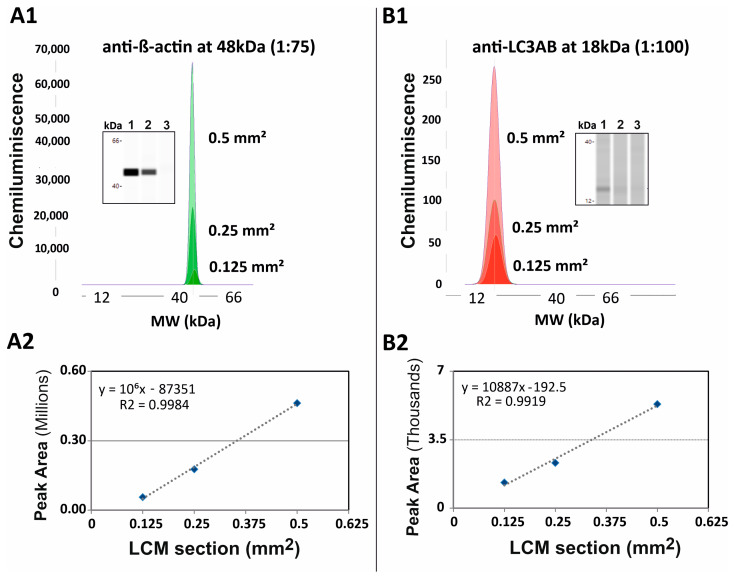
Cap-IA results of anti-β-actin (1:75) and anti-LC3AB (1:100) antibodies with three different LCM samples (0.5 mm^2^, 0.25 mm^2^, and 0.125 mm^2^) of rat kidney tissue. (**A1**) Electropherogram representation of cap-IA signals and gel-like image representation of cap-IA signals. Lane 1: 0.5 mm^2^; lane 2: 0.25 mm^2^; lane 3: 0.125 mm^2^ using anti-β-actin antibody. (**B1**) Electropherograms representation of cap-IA signals and gel-like image representation of cap-IA signals. Lane 1: 0.5 mm^2^; lane 2: 0.25 mm^2^; lane 3: 0.125 mm^2^ using anti-LC3 antibody. (**A2**,**B2**) Linear curve of areas under peaks in cap-IA (*y*-axis) versus loading protein amounts (*x*-axis) relationship showing decreasing intensities of bands or peak areas with a decreasing area of LCM samples.

For the optimization of anti-β-actin antibody dilution, five different dilutions of the anti-β-actin (1:25, 1:75, 1:100, 1:150, and 1:300) were used on a fixed LCM section of 0.5 mm^2^ area with 10 µm thickness from rat kidney. To select the best antibody dilution, only those dilutions which showed moderate baseline with high signal intensity were considered. The highest signal intensity was observed with 1:25 dilution but the baseline value of 1:25 dilution was higher than the signal of analytes in the other dilutions. In other words, such a high baseline may mask the signal of lower-abundant analyte and therefore may interfere with other antibodies during multiplexing. Keeping this point in mind, we have found that a dilution 1:75 of β-actin antibody was the best option for the assay, as well as for multiplexing with other antibodies (Figure 1). The signal of β-actin was observed at ~48 kDa, which was slightly bigger than the published molecular weight of β-actin (42 kDa; Figure 2A1). This setting was also tested with rat pancreas and heart.

To ensure the linearity of the signal from highly-abundant and low-abundance protein analyte, four different sizes of the dissected sample (0.5 mm^2^, 0.25 mm^2^, 0.125 mm^2^, and 0.06 mm^2^) were used from 10 µm-thick sections of rat kidney sections. Each sample was loaded onto three separate capillaries for the analysis. In the range from 0.125 mm^2^ to 0.5 mm^2^ of the LCM section area, we observed the linearity of signals for both β-actin (antibody dilution 1:75) and LC3A/B (antibody dilution 1:100). No signals were detected from the LCM section with an area of 0.06 mm^2^ (Figure 2).

**Figure 3:**
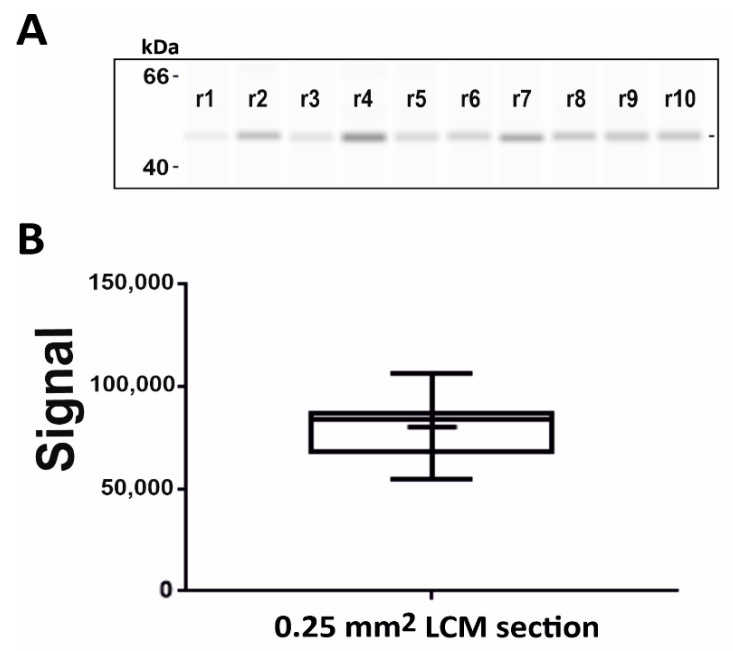
Reproducibility of LCM-cap-IA integration: Signal response of β-actin on 0.25 mm^2^ dissected LCM samples from rat kidney tissues with 10 µm thickness: (**A**) Gel-like image representation of cap-IA signals, (**B**) Data analysis of the area under the curve from 10 technical replicates, processed separately. Data were analyzed by box and whisker graph.

To ensure the reproducibility and accuracy of the signal, and to understand the manual error during sample processing, 10 technical replicates of 0.25 mm^2^ LCM sections of 10 µm thickness were run from rat kidney tissue (Figure 3). We added 8 µL of sample buffer on 0.4 mm^2^ LCM sections to maintain the final tissue area 0.05 mm^2^/µL. After sample processing, 5 µL of the sample was loaded to capillaries which were equivalent to 0.25 mm^2^ LCM sections. Each replicate was processed separately. The anti-β-actin antibody was used with dilution 1:75 for the analysis. The data were summarized by a box and whisker graph. The signal value for median was 83,759 and percentile values for 25% and 75% were 67,322 and 88,255, respectively. Moreover, reproducibility with a coefficient of variation of 18.5% was observed (Figure 3).

**Figure 4:**
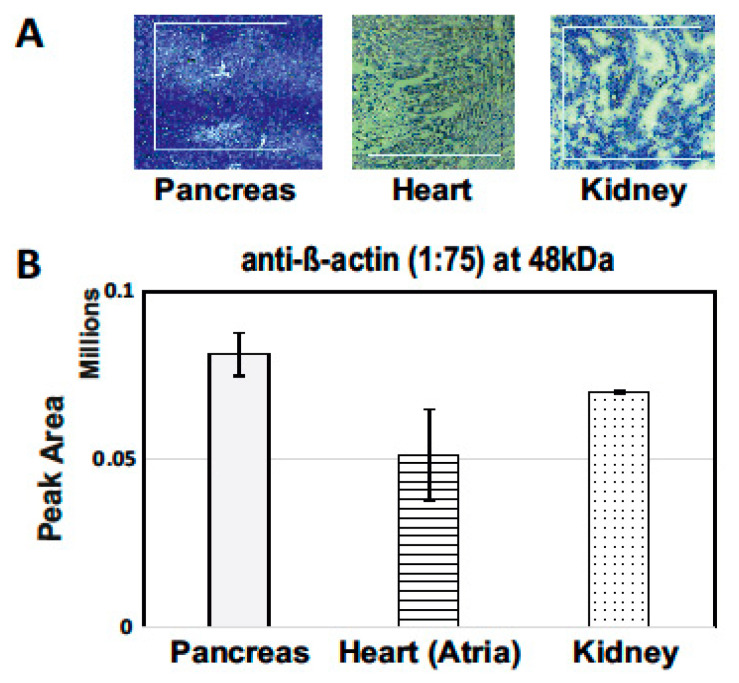
Signal response of β-actin on 0.25 mm^2^ dissected LCM samples from the pancreas, heart, and kidney tissues of rats: (**A**) Hematoxylin stained 0.25 mm^2^ of LCM section of 10 µm thickness were marked for cutting on microscope with laser at 10× resolution. (**B**) Data analysis of peak area under the curve.

To validate that the approach could be used with the tissues of different heterogeneity, the β-actin signal response from 0.25 mm^2^ LCM sections (10 µm thickness) from the kidney, heart, and pancreas of a rat was examined and compared (Figure 4). The highest β-actin signal response was observed from the pancreas and the lowest signal from heart tissue. The difference in the signal response was due to different heterogeneity among tissues. This clearly shows that prior optimization is required for using LCM-cap-IA integration (Figure 4).

### 3.2. Profiling of PCK2 in Langerhans Islets of Diabetic Rat

#### 3.2.1. Multiplexing of β-Actin with PCK2

**Figure 5:**
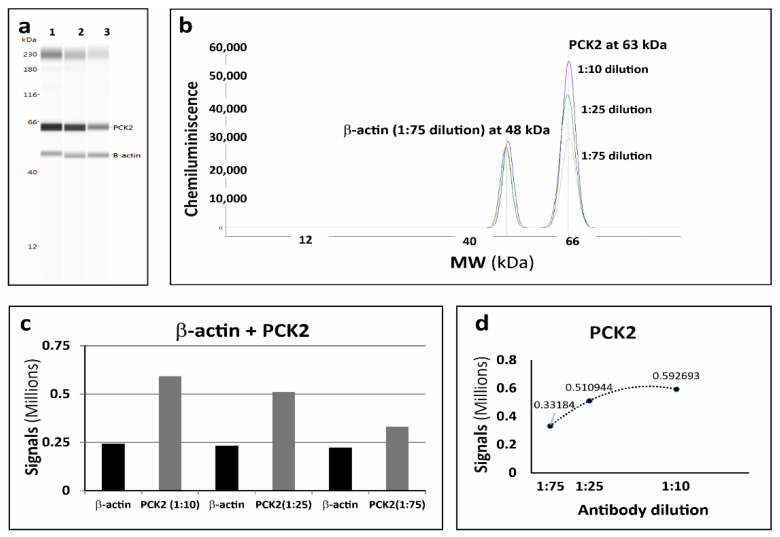
Multiplexing results of anti-PCK2 with anti-β-actin on capillary western blot. Three different dilutions of anti-PCK2 (1:10; 1:25; 1:75) were used with a fixed dilution of anti-β-actin (1:75) on a 0.5 mm^2^ LCM section of rat kidney tissue, with a thickness of 10 μm. (**a**) Gel-like image representation of cap-IA signals. Lane 1: anti-PCK2 dilution 1:10, lane 2: anti-PCK2 dilution 1:25, lane 3: anti-PCK2 dilution 1:75. (**b**) Electropherograms representation of cap-IA signals. (**c**,**d**) Data analysis of the area under the curve to understand the best dilution of anti-PCK2 for multiplexing.

Detection of more than one signal per cap IA capillary can enhance the throughput and reproducibility of the whole process. The multiplexing capacity of PCK2 antibody with anti-β-actin antibodies was analyzed on a fixed LCM section area of 0.5 mm^2^ with 10 µm thickness from rat kidney. Three different dilutions (1:10, 1:25, 1:75) of anti-PCK2 antibodies were mixed with the fixed dilution of the anti-β-actin antibody (1:75), separately. Three different mixtures were used separately and results were analyzed. Data analysis of areas under the curve was done to understand the best dilution of anti-PCK2 for multiplexing. It was observed that the dilution 1:75 of anti-PCK2 was the best suitable option for multiplexing with the anti-β-actin antibody. The signal of anti-PCK2 was observed at 63 kDa (Figure 5). 

#### 3.2.2. Detection of PCK-2 in Dissected Langerhans

**Figure 6:**
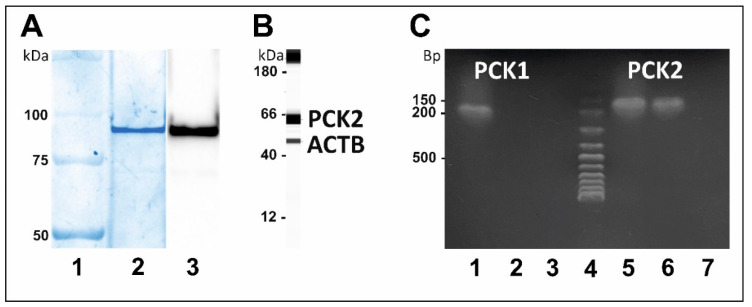
Identification of PCK-2 in dissected Langerhans. (**A**) Western blot analysis of recombinant PCK2: Lane-1: Marker; Lane-2: 1μg of recombinant PCK2 were examined onto 10% SDS-PAGE; Lane-3: Western blot analysis of recombinant PCK2 using anti-PCK2 antibodies (1:1000). We observed the immunogenic band of PCK2 at 96 kDa. (**B**) Capillary western blot of dissected Langerhans islets from rat pancreas using anti-PCK2 Abs (1:75). We observed the immunogenic band of PCK2 at 63 kDa. (**C**) Agarose gel electrophoresis of qPCR products of PCK1 and PCK2 amplified: Lane-1: kidney (+ve control); Lane-2: dissected Langerhans islets from rat pancreas; Lane-3: Water (-ve control); Lane-4: Marker; Lane-5: kidney (+ve control); Lane-6: dissected Langerhans islets from rat pancreas; Lane-7: Water (−ve control).

The commercially available recombinant PCK2 corresponding to the amino acids 1–640 of human PCK2 full-length was purchased and used to confirm the specificity of anti-PCK2 antibodies in conventional WB analysis. A single immunogenic band of PKC2 at 96 kDa was observed as mentioned in the datasheet of recombinant PCK2 (Figure 6A). The higher molecular weight of recombinant PCK2 was due to GST-tag at N-terminal but PCK2 corresponding to the amino acids 1–640 of human PCK2 full-length. In the capillary Western blot of dissected Langerhans islets, an immunogenic band of PCK2 at 63 kDa was visible using similar antiPCK2 antibodies (Figure 6A). Moreover, the presence of PCK2 was further validated by RT-qPCR analysis of RNA isolated from Langerhans islets. Product of appropriate size was obtained with primer pair specific to PCK2 mRNA. Additionally, RT-qPCR reaction with primer pair corresponding to PCK1 mRNA was done, in order to prove the production of this enzyme by the obtained cells. No specific product was detected in the LCM sample of the Langerhans islets with primer pair for PCK1 (Figure 6C).

#### 3.2.3. Expression of PCK2 in β-Cell Langerhans Islets of Diabetic Rat

**Figure 7:**
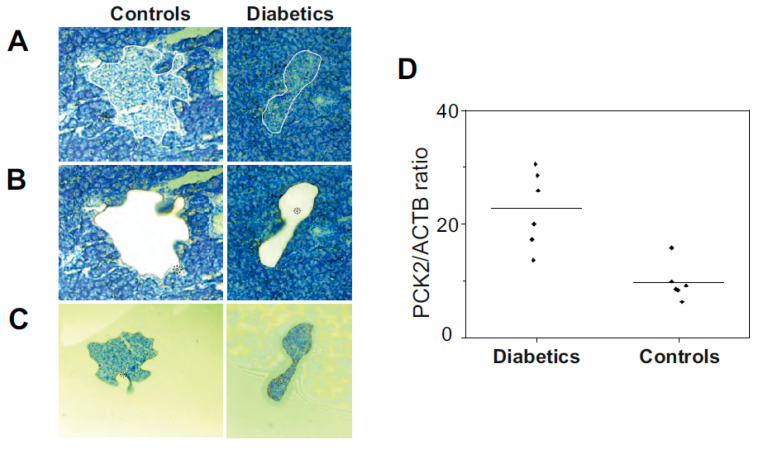
LCM dissection of Langerhans islets from rat pancreas: Hematoxylin-stained pancreatic tissue of 14 µm thickness on laser microscope at 10× resolution. (**A**) Langerhans islets were marked for cutting through laser. (**B**) Stained tissue sections after cutting of Langerhans islets. (**C**) Captured β-cells after laser cutting. (**D**) Capillary western blot of dissected Langerhans islets from diabetic rat (*n* = 6) versus control rat (*n* = 6). The signal ratio of PCK2/ACTB in diabetic and control groups (mean of technical replicates) was analyzed by *t*-test.

LCM-cap-IA integration was used to investigate the level of PCK2 in Langerhans islets of the diabetic pancreas. Frozen rat pancreatic tissues of diabetic rats (*n* = 6) and control rats (*n* = 6) were used for this study. Langerhans islets were dissected from a pancreatic tissue section of 14-µm thickness. For each rat, Langerhans islets were dissected until the total section area reached ≥ 0.8 mm^2^. Using the WES software (Compass for SW), peak areas of PCK2 peak (at 63 kDa) and ACTB peak (at 48 kDa) were extracted for each technical replicate. Normalization of PCK2 signal in each replicate was expressed as a ratio of PCK2 to ACTB peak areas (PCK2/ACTB). The average of PCK2/ACTB from three technical replicates was calculated for each animal (Supplementary Table S2). The difference between the healthy group and diabetic group was tested using the nonparametric *t*-test. The statistically significant difference between groups was found at a < 0.05 significance level. The study confirms the significantly higher expression level of PCK2 in diabetic Langerhans islets than normal Langerhans islets (Figure 7D).

## 4. Discussion

In recent years, considerable efforts have been made to enhance the overall sensitivity of immunoassays for quantifying proteins. For example, multiple antibody-based methods for quantifying proteins in single cells have been developed such as CyTOF [20,21,22], scWestern blots [11,23], and Proseek Multiplex, an immunoassay readout by PCR [24]. Moreover, newly-developed ultrasensitive protein assays like immuno-PCR could be seen as an incremental step in the field of immunological research and clinical diagnostics. The techniques are combined for nucleic acid amplification with an antibody-based assay which can dramatically increase the sensitivity of conventional immunoassays [25,26,27,28]. These methods can quantify up to a few dozen endogenous proteins recognized by highly specific antibodies and have enabled a lot many exciting research avenues. The decision regarding which method needs to be employed depends upon the special need of the biological or clinical question addressed and also necessitates a deeper exploration of the pros and cons of each method.

Immunoassays tend to be a more common technique that is routinely used in molecular biology and immunology labs. It is easily automated, requires less training, and can be easily integrated into the core laboratory facility. Immunoassays are based on primary antibody interaction with a specific target analyte in a complex mixture. Enzymes such as horseradish peroxidase (HRP) and AP are conjugated with primary/secondary antibodies to produce the signal. The reliability of immunoassays is majorly dependent upon the purity and specificity of primary and secondary antibodies. Therefore the throughput and accuracy of antibody-based methods are limited by epitope accessibility, and the availability of highly specific antibodies that bind their cognate proteins stoichiometrically [11], [29,30,31,32,33]. 

The main advantage of capIA is the processing of low sample quantities (sub microgram to nanogram). The possibility of automation can also reduce the technical variability and enables fast analyses. Multiplexing can further increase throughput. The speed of the process can be utilized in real-time diagnostics [34].

Proteomics utilizing LC-MSMS instrumentation represents an important possibility for the quantitation of protein analytes [35]. The development of methods such as NanoPOTS, SCoPE-MS has further revolutionized the implication of mass spectrometry (MS) in biological research [12,13], allowed to process samples in sizes of LCM section to a single cell.

The utilization of the LC-MSMS technique for the analysis of LCM sections represents its own challenges. Methods include solubilization by different buffers and overnight digestion. Variability in different protocols puts a demand for optimization and interpretation of results. Some procedures can also interfere with the LC-MSMS (e.g., HE staining reduces the signal in MS [5,36]. Optimization of analyte detection (in MRM mode), and longer time of analysis and data interpretation (in comparison with capIA) is also characteristic for LC-MSMS.

In this study, we have investigated a unique integration of LCM with cap-IA for obtaining a deeper and more accurate understanding of spatially defined regions of a complex heterogeneous tissue. LCM-cap-IA integration is a rapid, automated, and reliable approach for the analysis of proteins in dissected samples. In the cap-IA procedure using Wes^®^ instrument (ProteinSimple), the separation and immune detection steps take place in fully automated glass capillaries after sample loading. The sample is injected into the capillary and proteins are separated in stacking and separation matrix through electrophoresis. UV light cross-linking is used to immobilize the separated proteins to the wall of the capillary. Then capillaries are to be washed with buffer to remove the gel matrix and block with specific antibodies to provide a good signal-to-noise ratio [37,38]. On the contrary, membrane-based WB requires lengthy and time-consuming steps, which include sample loading, gel electrophoresis, transfer, blocking, primary and secondary antibody incubation, detection, and washing at each step. These steps are performed manually. Moreover, optimization of transfer of proteins to nitrocellulose or PVDF membrane is also critical to maximize the protein transferred to the membrane and minimize losses [39]. The cap-IA is rapid, automated, requires less time for sample preparation and plate loading is only ~2 h. The total run time of cap-IA is ~3 h, compared to ~20 h run time of membrane-based WB. The cap-IA setting employs primary and secondary antibodies in very low amounts (10 µL/ capillary) compared to conventional WB, which requires a very high amount (~3 mL to 5 mL/ blot) of primary and secondary antibody. These options make cap-IA, a better choice of integration for LCM dissected samples. Although the supply expenses are a bit higher in cap-IA, the offsetting advantages are obtaining higher quality of result, greater sensitivity, and time savings. These options make it a better choice of integration with LCM. The integration of LCM-cap-IA can be used to analyze the expression of proteins in limited content of specific cell populations embedded within heterogeneous cell populations of the tissue. For example; the study of neurons in the heart and kidney or the study of β-Cells in the pancreas etc. The above-mentioned integration of LCM-cap-IA will further support the genomic data obtained from the RT-qPCR analysis of the LCM dissected sample.

The quantitative multiplex analysis is the detection of signals from more than one antibody in the same capillary. For developing a good multiplexing methodology, careful optimization is required. However, already published experiments utilize cap-IA without multiplexing, which means only one antibody per capillary and housekeeping antibodies in separate capillaries [40,41]. Therefore, the possibility of multiplexing can further enhance the throughput of cap-IA but the challenges are: (1) targeted protein analytes should be in the same linear range of detection; (2) molecular weight of target proteins should be distant from each other to show good resolution of peaks/bands; (3) baseline of the individual antibodies should be minimum; (4) host of the two or more different primary antibodies should be same if they are in the similar linear range of detection and host should be different if they are in the different linear range of detection.

The integration of LCM-cap-IA methodology is less demanding, showing high reproducibility and allowing multiplexing. In our experiment, we have observed linearity of the signal with *R*^2^ > 0.9 for both ranges of proteins high to low abundance protein i.e., β-actin, LC3AB, respectively (Figure 2). However, the main challenge was to detect low-abundance metabolic protein in microdissected sections such as 0.125 mm^2^, because the signal from the target protein may be masked by a very high baseline response of housekeeping antibody.

For successful multiplex analysis, it is important to maintain the baseline value of antibodies in the range, which does not mask the signal of each other. On the contrary, un-optimized integration of LCM samples with cap-IA is difficult due to the low protein amount, was not easy to handle. We have also observed that LCM section area ≥0.250 mm^2^ with thickness 10 um had more chances to provide good reproducibility data with multiplexing for cap-IA analysis. Surprisingly, we have seen some articles for integration of LCM with qPCR for transcriptome profiling, but have not found any publications, which combine LCM to cap-IA for proteomic study. Thus, our LCM-cap-IA integration was applied to study the impact of diabetes on the expression of PCK2 within the Langerhans islet.

The importance of PCK in the pathophysiology of diabetes has been studied by several researchers, focusing mainly on PCK1, a cytosolic isoform of this enzyme. It has been reported that the overexpression of PCK1 in type 1 diabetes mellitus leads to increased gluconeogenesis in the liver and kidney [42], insulin resistance [43], and hyperglycemia [44]. Subsequently, PCK1 gene silencing has a direct impact on glycemic control and energy metabolism [45]. Interestingly, pancreatic β-cells do not express PCK1 but only PCK2 [15,46]. PCK1 has been shown to be present within α-cells of Langerhans islets localized on the surface of the islets [19].

PCK2 is a mitochondrial isoform of PCK, which, as well as PCK1, behaves as a gluconeogenic enzyme. It acts as a “metabolic tachometer” to sense TCA cycle flux and regulates glucose homeostasis by modulating the production and clearance of glucose. Under the hypoglycemic condition, PCK2 allows cells to produce PEP from oxaloacetate, which is a requirement for gluconeogenesis from mitochondrial substrates. Therefore, PCK2 can also regulate gluconeogenesis under a nutrient-deficient environment through decarboxylation of oxaloacetate to PEP in the tricarboxylic acid (TCA) cycle. Mitochondrial PEP synthesis is coupled to insulin secretion [15]. Additionally, PCK2 is involved in the regulation of lipid metabolism, while overexpression of PCK2 can lead to lipid deposition in cells [47]. Based on this evidence, the role of PCK2 in the pathophysiology of diabetes could be suggested. However, to date, research on this enzyme with diabetes mellitus has focused primarily on PCK1 isoform.

Expression and protein level of PCK2 has been studied in hepatocytes of chronically glucose-infused rats. Elevated plasma level of glucose and insulin was accompanied by increased expression of PCK2 on genomic as well as the proteomic level [48]. Islets predominantly consist of insulin-producing β-cells and glucagon-producing α-cells, and other cells such as δ-cells, γ-cells, and ε-cells. Diverse patterns of endocrine cell arrangement was seen in different animals. In normal rodent islets, β-cells forming the core surrounded by other endocrine cells in the periphery [49]. In this study, attention was placed on Langerhans islets mainly for β cells whereas α-cells were omitted during laser cutting of border area for each Langerhans islet [19]. which was further confirmed by qPCR analysis of PCK-1 and PCK-2. In the dissected Langerhans, we have only observed the gene expression for PCK-2, which confirms the predominance of β-cells in dissected samples (Figure 6C).

To analyze a comparable amount of β cells in each sample, Langerhans islets were microdissected by LCM from sections of pancreatic tissue of both, control and diabetic, animals. Model of long-term type 2 diabetes was used to detect the availability of PCK2 in the situation when a substantially lower amount of insulin is synthesized by this type of cells [50]. The results show that PCK2 expression at the proteomic level is significantly increased in the islets of Langerhans, indicating the persistence of PCK2 in cells, although it cannot fulfill one of its most important functions, regulating insulin release, suggesting its role in the pathophysiology of type 2 diabetes mellitus. Additionally, it was demonstrated that PCK2 up-regulation is a novel metabolic adaptation in cancer [51]. From these findings, it can be concluded that PCK2 is up-regulated in Langerhans islets of T2D rats, which, together with information from the literature, suggests its role as an important regulatory factor of cellular metabolism involved in various pathological conditions, also including diabetes.

## Supplementary Materials

The following are available online at https://www.mdpi.com/article/10.3390/pharmaceutics13060883/s1, Figure S1: Hematoxylin stained rat kidney tissue of 10 μm thickness; Figure S2: Calibration curve for protein estimation of LCM sample Table S1: Estimation of protein amount in LCM sections, Table S2: Normalized values of PCK2 in β-cells of diabetic and control groups for each animal.

## Abbreviations

LCM	Laser capture microdissection
cap-IA	capillary-based immunoassay
RT-qPCR	reverse transcription followed by quantitative real-time polymerase chain reaction
NGS	Next-generation sequencing
PCK2	mitochondrial phosphoenolpyruvate carboxykinase
ACTB	beta-actin
WB	Western blotting
