# Supplementary material for: Laser Capture Microdissection Coupled Capillary Immunoassay to Study the Expression of PCK-2 on Spatially-Resolved Islets of Rat Langerhans
Source: Pharmaceutics. 2021 Jun 15;13(6):883. doi: 10.3390/pharmaceutics13060883 (PMC8232303; doi:10.3390/pharmaceutics13060883)
Supplement: Supplementary file 1 [file pharmaceutics-13-00883-s001.zip › pharmaceutics-1232955-supplementary.pdf]

pharmaceutics

MDPI

Supplement Materials: Laser Capture Microdissection Coupled Capillary Immunoassay to Study the Expression of PCK-2 on Spatially-Resolved Islets of Rat Langerhans

Shashank Pandey, Zdenek Tuma, Tereza Smrhova, Miroslava Cedikova, Tereza Macanova and Magdalena Chottova Dvorakova

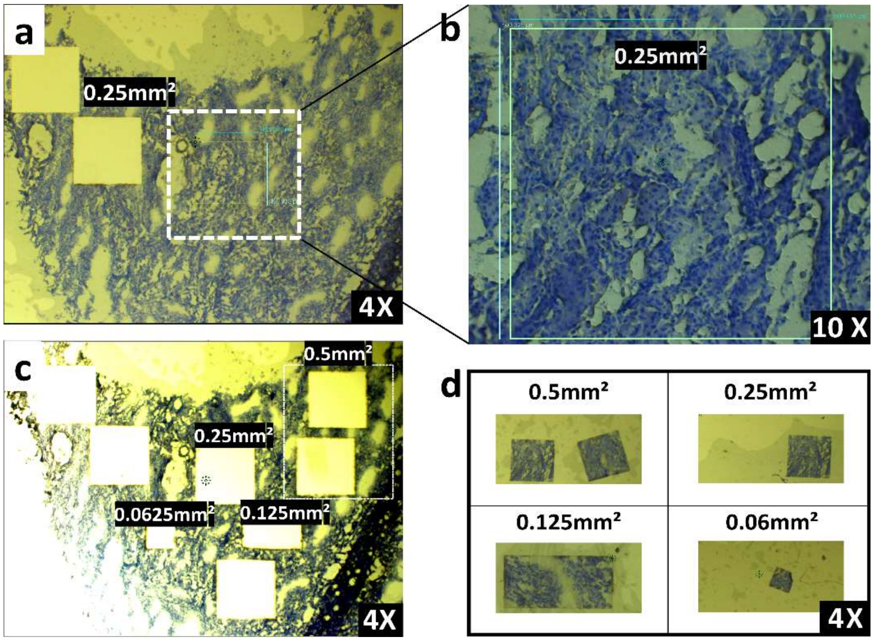

Figure S1. Hematoxylin stained rat kidney tissue of 10 μm thickness. (a) 0.25 mm^2 of tissue sections marked for cutting through laser at 4× resolution. (b) 0.25 mm^2 of stained tissue sections marked for cutting through laser at 10× resolution. (c) Stained tissue sections after cutting of all the sections (0.5 mm^2, 0.25 mm^2, 0.125 mm^2 and 0.0625 mm^2) at 4× resolution. (d) Tissue samples captured after laser cutting.

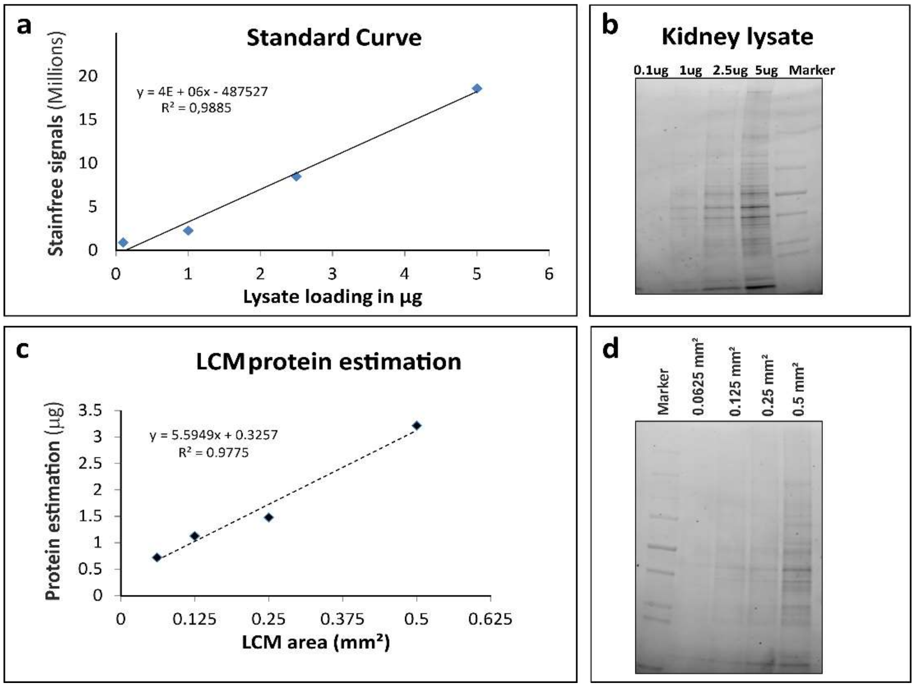

Figure S2. Calibration curve for protein estimation of LCM sample. (a) Rat kidney lysate was used to prepare the calibration curve. Calibration curve was linear with ($R^2 = 0.98$). (b) Rat kidney lysate proteins of known concentration (0.1 µg, 1 µg, 2.5 µg and 5 µg were examined onto 10% SDS- PAGE (Stain free gel, Bio-Rad). (c) Linear curve of proteins extracted from different LCM samples of rat kidney (0.5 mm^2; 0.25 mm^2; and 0.125 mm^2). Protein estimation in µg (y axis) versus LCM area sections (x axis). Calibration curve was linear with (R^2=0.98). (d) Proteins extracted from LCM samples of rat kidney with different areas (0.5 mm^2, 0.25 mm^2 and 0.125 mm^2) were examined onto 10% SDS- PAGE (Stain free gel, Bio-Rad) and presented at **Table S1**.

Table S1. Estimated total protein amount for each LCM section area.

LCM section area (mm^2)	Protein amount in section (µg)
0.0612	0.597
0.1250	0.658
0.2500	1.412
0.5000	2.875
1.0000	3.074

Table S2. Normalized values of PCK2 in β-cells of diabetic and control groups for each animal.

Animals	Average of Signal Ratio (PCK2/ACTB)	
	Diabetic	Control
1	28,51706	9,882957
2	17,29695	8,59383
3	30,46167	15,78045
4	25,86456	9,16841
5	13,64311	8,410773
6	19,91703	6,295529
